# Supplementary material for: Are carbon emissions trading and green financial instruments synergistic? -Comprehensive quantitative research based on content analysis
Source: PLoS One. 2024 Mar 7;19(3):e0298601. doi: 10.1371/journal.pone.0298601 (PMC10919601; doi:10.1371/journal.pone.0298601)
Supplement: S1 Data — (ZIP) [file pone.0298601.s001.zip › Data/地级市Z_F工作报告.zip!/║■▒▒/╛ú╓▌╩╨/└┤╫╘╣1⁄2╓┌║┼í╛┬φ┐╦╩2╛▌═°í┐_2009.docx]

各位代表：

现在，我代表市人民政府向大会作工作报告，请予审议，并请市政协各位委员和列席人员提出意见。

一、2008年工作回顾

2008年是极不平凡的一年。在省委、省政府和市委的正确领导下，在市人大、市政协的监督支持下，市政府认真贯彻落实科学发展观，团结带领广大干部群众，抢抓机遇，克难奋进，全市经济社会发展呈现增长较快、质量提升、后劲增强、民生改善的良好态势。全年生产总值624亿元，增长12.6%；固定资产投资291.5亿元，增长38.3%；社会消费品零售总额370.6亿元，增长23.7%；出口6.15亿美元，增长26.2%；实际利用外资1.17亿美元，增长16.7%；财政收入42亿元，增长23.3%，其中地方一般预算收入18.7亿元，增长22.7%；城镇居民人均可支配收入12195元，增长12.5%；农民人均纯收入4889元，增长18.1%;居民消费价格上涨6.4%；城镇登记失业率控制在4.4%以内；人口自然增长率为4.3‰；万元生产总值能耗和化学需氧量、二氧化硫减排目标任务完成。除居民消费价格指数外，市三届人大四次会议确定的主要目标任务全面完成，为实现“十一五”规划奠定了坚实的基础。

积极应对复杂经济形势。过去的一年，政府工作面临前所未有的严峻考验。在抗击年初50年一遇的冰雪灾害中，全力以赴，众志成城，保电力、保交通、保供应、保民生，争取救灾资金4797万元，提前完成雪灾倒房恢复重建任务。在“5.12”汶川特大地震发生后，情系灾区，踊跃捐助，积极开展对口援建，募集救灾款物8190万元。在国际金融危机影响逐步显现，部分行业、企业生产经营困难的严峻形势下，沉着应对，因企施策，及时出台扶持纺织行业发展的政策措施，积极解决突出困难和问题，努力保障经济健康运行。在举世瞩目的奥运火炬荆州站传递活动中，精心策划，周密部署，圣火传递安全有序、精彩纷呈、圆满成功，有力提升了荆州的知名度和影响力。在金融危机影响持续加深的大背景下，主动出击，借力造势，成功举办第二届全球华商500强高科技论坛暨荆州经贸洽谈会，拓展了扩大开放和承接产业转移的重要平台。在抢抓国家宏观政策调整和增发国债的机遇过程中，加强对接，积极运作，争取和落实中央新增投资5.1亿元，居全省第二位。期盼多年的“两大铁路”--汉宜高速铁路荆州段全面开工建设，荆岳铁路有望今年年底前开工；“两条高速”--洪监高速公路可望今年9月前开工，江南一级公路规划调整为高速公路；“四座电厂”--国电长源2×30万千瓦首台机组将于今年9月投产，沙隆达自备电厂今年将开工建设，楚源自备电厂、荆沙发电厂2×60万千瓦项目预可研通过评审；荆南四河堤防整险加固、四湖流域综合治理工程取得突破性进展，总投资25亿元的部省联合防治血吸虫病项目正式启动。这一大批重大项目的实施，必将极大地改善我市发展条件。

经济保持较快增长。深入实施工业兴市战略，工业发展质量和后劲增强。规模以上工业完成增加值173.9亿元，增长24.5%，超过全省平均水平2.9个百分点；规模以上企业净增112家，达到901家；完成工业投资134亿元，工业入库税金19.1亿元，拉动财政收入增长9.4个百分点。“福娃”、“白云边”和“雪尔”跨入“中国驰名商标”行列。中心城区规模以上工业增加值89.6亿元，占全市的51.5%。工业园区完成基础设施投资4.9亿元、入园企业和项目232个，其中荆州开发区引进千万元以上项目61个、建成投产62个。加快推进农业产业化经营，农业综合生产能力稳步提高。粮食产量连续五年增产，达到344.5万吨，增25.4万吨；棉花总产15万吨，减0.67万吨；油菜总产48.4万吨，增3.6万吨；生猪出栏358.4万头，增42万头；家禽出笼4763万只，增1035万只；水产养殖面积达到188万亩，产量84.3万吨、增15.4万吨。新增市级以上龙头企业32家、农民专业合作社124家、标志性农产品64个。安全饮水、农村沼气、土地整理等项目建设力度加大，仙洪新农村试验区建设全面展开，建成通村公路1442公里，新建沼气池6万口，新增安全饮水人口45.9万人，完成新农村建设投资40亿元以上。大力发展文化旅游和商贸物流业，现代服务业活力增强。稳步推进旅游项目建设，熊家冢考古发掘与文物保护工作取得进展，旅游专用公路顺利动工，张居正故居一期工程和关羽祠复建项目完工，洪湖乌林温泉国际度假区开工建设，北京专场旅游推介活动取得成效，全年接待境外游客2.89万人次、增长4.3%。加强商贸物流市场建设，新建和改造农家店714家，城乡消费更加活跃。整顿规范烟草市场，烟草销售实现利税5.06亿元。积极拓展金融服务，邮政储蓄银行荆州分行、市商业银行松滋支行和湖北公安花旗银行贷款公司挂牌成立，实现省“A级金融信用市州”创建目标。金融机构存款余额730亿元，累计信用融资总量达到290亿元。

改革开放取得新进展。深入推进农村综合改革，“以钱养事”新机制进一步完善，集体林权制度改革稳步推进，土地流转试点工作顺利开展。强化国有资产监督管理，国有存续企业资产打包处置和非工企业改革步伐加快，天发集团风险处置取得重大突破，天颐科技成功恢复上市。实行规范的市直部门预算和公务卡结算方式，收支两条线、非税收入管理得到加强。完善中心城区招投标“管招分离、同城合一”工作机制，招投标市场更加规范。调整中心城区城管体制，下放管理事权，调动基层参与城市管理的积极性和主动性。启动事业单位分类改革，水利工程管理体制改革全面完成。深化行政审批制度改革，减少审批项目163项、精简35.4%，经济发展环境进一步优化。狠抓园区招商、驻点招商，招商引资成效显著，到位资金保持大幅增长。新批外商投资企业15家。海关、出入境检验检疫、口岸等大通关建设得到加强，盐卡口岸集装箱吞吐量达到5.3万标箱，增长2%。

城乡建设步伐加快。交通完成投资23.3亿元，东荆河大桥主体工程完工，盐卡二期码头水工部分建成，宦子口船闸竣工验收，随岳高速荆州段、荆监一级公路、荆岳长江大桥等工程建设进展顺利。水利完成投资7.4亿元，高潭口、黄山头等5处大型泵站更新改造完工，水库整险、大型灌区续建配套与节水改造工程完成年度任务，四湖流域综合治理、荆南四河整险加固等重点工程建设顺利推进。电力、电信分别完成投资16.69亿元和1.85亿元。中心城区城市总规修编工作通过部级评审，城市建设完成投资4亿元，荆中路、屈原路、郢都路、南环路、东环路和塔桥路实现黑化升级，荆沙大道全线贯通，武德路卡口打通，荆襄河、荆沙河清淤和四湖流域污水治理等水环境整治工程全面展开，市容市貌较大改善。房地产市场平稳发展，完成投资26.6亿元，增长19.8%。县市城区和中心镇建设力度加大，全市城镇化率达到36.6%。

社会事业协调发展。着力建设创新型城市，争取省以上科技项目79项、资金2490万元。建立健全义务教育经费保障机制，补助各类资金3.4亿元，受益学生65万人。进一步完善农村和社区卫生服务体系，建设农村标准化卫生室344个；加快实施血防整县推进和综合治理工程，救助晚血病人3048人，血吸虫病疫情控制达标工作顺利通过国家验收。深入开展群众性精神文明创建活动，公民思想素质和城乡文明程度进一步提高。繁荣发展文化事业，乡镇综合文化站、农村电影放映等文化惠民工程投入资金1500万元，数字电视整体转换工程全面启动，荆州博物馆获评国家一级博物馆并免费开放。广泛开展全民健身运动，荆州健儿江福英勇夺北京残奥会金牌。强化计生优质服务，人口和计划生育工作进一步加强。扎实开展节能减排专项治理，关闭小印染企业15家，小水泥、小炼钢等企业7家。加大基本农田保护力度，土地整理新增耕地2.1万亩。深入开展严打整治斗争，强化治安防控体系建设，我市被评为全省社会治安综合治理优胜单位，通过全国社会治安综合治理优秀市检查验收。加强法律援助工作，建成市级法律援助远程网上申请、受理、咨询系统。广泛开展大接访活动，一批信访疑难问题得到妥善解决。深入开展“隐患治理年”活动和百日督查专项行动，强化食品、药品质量监管，重特大安全事故得到有效遏制，被授予全省安全生产红旗单位。第二次经济普查全面展开。加强国防教育、国防动员、人民防空、预备役部队和民兵建设，认真做好转业退伍军人安置和拥军优属工作，军政军民关系进一步密切。妇女儿童和未成年人、老年人、残疾人的合法权益得到保护。外事侨务、台湾事务、民族宗教、社会科学、盐业、邮政、气象、地震、档案、史志和无线电管理等工作都取得了新成绩。

人民生活进一步改善。认真落实惠农政策，发放农业补贴资金6.6亿元，比上年增加2.9亿元；农民负担总额1.28亿元，比上年减少1亿元。扎实开展创业促就业全民行动，新增城镇就业人员9.4万人，帮助3140人实现创业，带动1万多人就业；培训农村劳动力5.5万人，转移农村劳动力15.3万人。社会保险扩面新增9.7万人次，征收社保费23亿元。新型农村合作医疗基本实现全覆盖，参保人数达到359万人；城镇居民医疗保险试点工作扎实推进，登记参保人数达到85万人。城乡享受低保人数分别达到14.5万人、14.8万人，发放低保资金2.8亿元。中心城区开工建设经济适用房9.5万平米，对3294户低收入家庭发放住房困难补贴373万元。免费为100名贫困下肢残疾人安装假肢，为100名贫困白内障患者实施复明手术，对105户农村贫困残疾人危房改造实施补助。投入扶贫开发资金1.28亿元，2.4万人实现脱贫。投入移民扶持资金3500万元，移民生产生活条件逐步改善。市政府年初承诺的十件实事，除城南、草市污水处理厂完成主体工程、尚未投入运行外，其他事项全面完成。

认真执行市人民代表大会及其常务委员会的决议，主动接受人大及常委会的法律监督和工作监督，并就重大问题与政协民主协商，接受人民政协和各民主党派、工商联、人民团体、无党派人士的民主监督，接受新闻舆论和社会公众的监督，办理人大代表建议和议案138件、政协委员提案248件。积极推进基层民主政治建设，村委会换届选举顺利完成。集中开展行政执行力大讨论和文明执法教育两项活动，推行行政审批“两集中、两到位”改革，加强基层便民服务中心建设，行政服务效率进一步提高。深入开展行风评议和绩效考核，强化行政监察、审计监督、政务督查和政务公开，强化决策咨询、电子政务、新闻发言人制度和应急机制建设，服务型政府建设得到加强。

各位代表！过去一年取得的成绩，是上级党委、政府和市委正确领导，全市人民团结拼搏、共同努力的结果。在此，我代表市人民政府，向为荆州改革发展稳定做出积极贡献的广大干部群众，向全市人大代表、政协委员、各民主党派、工商联、人民团体和无党派人士，向驻荆部队、武警官兵和政法干警，向所有关心支持我市经济社会发展的各界朋友，表示衷心的感谢并致以崇高的敬意！

在看到成绩的同时，我们也清醒地认识到，发展不快、发展不够仍然是荆州最大的实际，经济社会发展中还存在不少矛盾和问题。主要表现在：制约科学发展的体制机制障碍依然存在，改革创新意识亟待增强；工业经济、县域经济、民营经济实力不强，结构调整和转变发展方式的任务艰巨；农业基础脆弱，农产品价格波动和农民工大量返乡，农民持续增收的难度增加；就业、社会保障、医疗卫生等民生问题尚未得到很好解决，部分群众生活困难；社会治安、城市管理、市场秩序还存在不少薄弱环节，影响社会稳定的因素依然较多；少数政府部门工作人员服务意识不强，办事效率不高，投资环境需要进一步优化；办理人大代表、政协委员建议提案的质量有待提高，政府执行力和自身建设有待加强。对此，我们将高度重视，采取切实有效措施，认真加以解决。

二、2009年政府工作的总体要求

2009年是建国60周年的喜庆之年，是实施“十一五”规划的攻坚之年，是深入开展学习实践科学发展观活动、促进科学发展上水平的重要一年。做好今年的工作，意义十分重大。

今年政府工作的首要任务是保增长。只有保持较快增长速度，才能够缩小发展差距，才能够更好地解决就业等民生问题和化解各类社会矛盾。实现保增长，我们面临着前所未有的困难和挑战。国际国内经济增速减缓，市场需求约束加剧，部分企业产销下滑，经济发展、社会稳定的压力加大；财政减税让利政策和改善民生刚性支出增多，收支平衡压力加大；社会投资趋于谨慎，扩大投资压力加大。在困难和挑战面前，坚定信心是重要前提，科学发展是根本出路，真抓实干是有效保证。危机中存在商机，挑战中蕴藏机遇。应该看到，实现保增长的有利因素很多，机遇前所未有，机遇大于挑战。一是党的十七届三中全会就推进农村改革发展作出重大部署，有利于我们破解“三农”难题，规划投资30亿元的仙洪新农村试验区和投资25亿元的血防部省联动项目及四湖流域综合治理建设步伐加快，必将带动我市农村加快发展。二是国家实施一系列扩大内需、促进经济增长的政策措施，加强对粮食主产区、中西部地区的基础设施、社会事业、社保民生等方面的投入，将为实现平稳较快发展提供坚强支撑，有利于我们改善基础设施条件，增强发展活力和后劲。三是省委、省政府深入实施“一带两圈”战略，荆州在全省经济发展中的地位和作用更为重要。加快推进汉宜、荆岳铁路和江南、江北高速公路建设步伐，为把我市建设成国家交通枢纽型城市、长江经济带重要节点城市奠定了基础；实施鄂西生态文化旅游圈发展战略，我市历史文化资源优势凸显，为推动文化旅游产业发展带来了新的重大机遇。四是历届市委、市政府“打基础、管长远”积蓄的发展能量不断释放，广大干部群众团结一心促发展、共克时艰保增长的愿望强烈，为我们积极应对和抵御风险提供了坚实的物质基础和强大的思想保障。实现保增长，必须坚定信心，既要清醒地看到国际国内经济形势的复杂性和严峻性，又要充分认识具备的有利条件和存在的发展机遇，从最坏处着想，向最好处努力，努力变困难为动力、化挑战为机遇，牢牢把握保增长的主动权。必须抢抓机遇，认真研究国家政策导向，用好用足国家政策，坚持扩投资、促消费、稳出口的工作思路，落实好保增长、调结构、重民生的具体措施，进一步增强践行科学发展观的自觉性，推进改革开放的坚定性，促进社会和谐的主动性。必须真抓实干，始终以昂扬向上的精神状态，把克服当前困难与促进长远发展有机结合起来，充分利用困难和危机形成的“倒逼”机制，抓紧解决经济发展中存在的深层次矛盾和问题，为今后更长时间、更高水平发展打下坚实基础。必须因势利导，在推进基础设施建设、承接产业转移、强化企业服务、加快技术改造和经济结构调整等方面狠下功夫，在困难时期以特殊之为扎实做好各项工作，确保经济在较快增长区间正常运行。

今年政府工作的指导思想是：认真贯彻党的十七大、十七届三中全会精神，以邓小平理论和“三个代表”重要思想为指导，全面落实科学发展观，把保增长作为首要任务，抢抓发展机遇，积极应对挑战，着力扩大内需转变发展方式，调整经济结构提升发展质量，深化改革开放增强发展活力，注重改善民生解决热点难点问题，促进经济社会又好又快发展。

经济社会发展的主要目标是：生产总值增长11%，固定资产投资增长25%，社会消费品零售总额增长16%以上，外贸出口增长12%，实际利用外资增长12%，财政一般预算收入增长13%；城镇居民人均可支配收入增长8%，农民人均纯收入增长7%，居民消费价格涨幅控制在4.5%以内，城镇登记失业率控制在4.5%以内，人口自然增长率控制在6‰以内，万元生产总值综合能耗、化学需氧量排放、二氧化硫排放分别下降4.8%、3.3%和1.4%。在实际工作中，我们将本着“跳起来摘桃子”的精神，争取实现更高的目标。

三、积极应对挑战，保持经济平稳较快增长

坚持把增加投资和扩大内需作为保增长的根本途径，把结构调整和转变发展方式作为保增长的主攻方向，把深化改革、扩大开放作为保增长的强大动力，推动经济持续健康发展。

（一）扎实开展项目建设年活动，千方百计扩大投资。积极争取中省投资和扩大招商引资，着力加强工业、水利、交通、城建等项目建设，努力发挥项目促投资、保增长的重要作用。

突出抓好重大项目建设。强化抓项目就是抓发展的意识，抓住国家扩大内需、增加投资和实施十大产业振兴规划的难得机遇，继续狠抓一批打基础、管长远的重大项目，狠抓一批见效快、效益好的工业、物流、旅游项目，狠抓一批教育、卫生、文化、体育、社会保障、生态建设等民生项目，努力形成“大投入、大建设”的发展格局。进一步加大项目前期工作力度，提升项目规划和可研水平，千方百计争取更多项目。继续实行领导联系重大项目制度，努力解决项目融资、用地等实际困难，切实加快项目建设进度。水利重点抓好荆南四河堤防、病险水库加固、第二批大型泵站改造和四湖流域综合治理等工程，积极争取荆江大堤综合治理、长湖洪湖整治和水利血防等项目。交通重点建成洪湖东荆河大桥、随岳高速荆州段、荆监一级公路和盐卡码头二期工程，加快荆岳长江大桥建设进度，开工建设洪监高速，力争江南高速和荆州港危化品码头动工建设。继续做好项目征地拆迁和协调服务，推动汉宜铁路、荆岳铁路和引江济汉等项目加快建设步伐。电力重点抓好国电长源热电项目投产、沙隆达自备电厂开工和城市生活垃圾焚烧发电等项目建设。在加快落实“十一五”规划项目的同时，争取更多项目增补进国家、省产业发展等专项规划和部省联动项目规划。充分发挥政府投资导向作用，放宽项目准入条件，鼓励和引导民间资本扩大投资。

突出抓好招商引资工作。依托产业特色、区位条件和资源优势，做好项目策划等基础工作，主动承接产业转移，做大做强主导产业。发挥企业招商主体作用，创新招商方式，推行园区整体招商、代理招商、驻点招商和回归招商，提高招商实效。精心组织长三角、珠三角等招商活动，充分利用深圳高交会、中博会等平台，着力引进一批技术含量高、投资强度大、产出效益好的项目。强化签约项目跟踪服务，完善督办考核机制，推动项目尽快落地，力争到位资金增长15%。

突出抓好城镇建设和管理。中心城区以创建省级卫生城市为目标，进一步完善基础设施，改善市容市貌，提升城市品位，增强综合实力和带动能力。抓紧编制荆州火车站和开发区综合服务中心等片区控制性详规，严格规划管理，维护规划的权威性。重点完成荆沙大道建设和北京路、红门路改造等工程，狠抓城区绿化配套，实施城区水域连通工程，切实改善城区水环境。做实做大城投公司资产，强化土地收购储备，增强融资功能，保障城市建设资金需求。着力整治小街小巷和城乡结合部脏乱差、集贸市场占道经营、乱搭乱建等突出问题，规范道路交通秩序，全面提高城市管理水平。加快城区危旧房改造步伐，完成喜雨街、绿化村危房改造主体工程，引导房地产业健康发展。同时，加强重点镇建设，强化产业支撑，健全配套服务功能，不断提高城镇化水平。

（二）加快新型工业化步伐，提升工业总量和质量。坚定不移地实施工业兴市战略，深入推进满园工程，壮大工业规模和产业集群，提升工业发展水平，力争规模以上工业增加值增长15%以上。

着力服务企业发展。树立“产业第一，尊重企业”的观念，动员社会各方面服务企业，支持企业。扩大中小企业发展专项资金规模，积极筹措工业生产调度资金，大力支持工业企业发展。认真落实增值税转型改革和停征部分行政事业性收费等扶持政策，减轻企业税负。强化银企合作和中小企业融资担保服务，建立重点企业直通车服务制度，千方百计帮助企业解决实际困难。坚持因企制宜，分类指导，支持中小企业与大企业大集团协作配套，转型升级，促进上规模、上水平。积极引导企业强化管理，向内挖潜，开拓市场，提升整体素质和市场适应能力。

着力培育市场主体。继续实施双百工程，对产销过亿、税收过千万的重点企业实行跟踪服务，支持一批基础较好和盈利能力较强的企业做大做强，扶持一批具有技术和产品优势的企业加快发展，帮助一批市场潜力较大和吸纳劳动就业能力强的困难企业搞活经营；对投资过千万的重点工业项目实行动态管理，抓好沙隆达吡啶、奥达纺织、宝利多晶硅、白云边新生产基地、新生源L-亮氨酸等项目开工建设和投产见效。认真抓好重点技术改造项目的筛选、储备，积极申报和争取技改专项，对成长性好、有发展后劲的优质技改项目给予资金扶持。加快实施中小企业成长工程，引导中小企业朝“专精特新”方向发展，力争全市规模以上企业达到1000家以上。

着力发展产业集群。荆州开发区着力抓好“五园两中心”建设，推进深圳电子工业园、纺织印染循环经济工业园项目入园。抓好工业园区产业规划、基础设施和配套服务设施建设，提高园区规划、管理和服务水平，推进企业布局集中和产业集聚发展，加快形成产业规模优势和特色品牌优势。抓住沿海产业转移步伐加快以及资本并购重组活跃等有利条件，加快机械汽配、电子信息、纺织印染、化工医药、石油装备、食品加工和森工造纸等产业集群建设，不断提高产业集中度和综合配套能力。用足用好扶持县域经济发展的政策措施，抓好产业布局整合和引导，壮大县域经济规模和实力。力争全市工业园区入园企业和项目200个以上、建成投产150个以上；荆州开发区新引进过千万元项目60个以上，新投产项目60个以上，在建项目60个以上。

着力提高创新能力。积极运用高新技术改造提升传统产业，大力扶持电子信息、新材料、生物医药等产业发展，提升高新技术产业比重。强化企业创新主体地位，支持企业创建研发机构，开展产学研对接，提高科技成果转化和产品研发能力。积极推进质量振兴活动，支持有竞争优势的产品争创品牌、抢占市场，扩大与知名品牌企业的合作，不断提高品牌产品的市场份额和竞争力。高度重视企业家队伍建设，大力引进和培养企业经营人才，进一步营造关心、爱护企业家的良好氛围。

（三）积极发展现代农业，扎实推进新农村建设。认真贯彻落实党的十七届三中全会精神，把建设社会主义新农村作为战略任务，把发展现代农业作为基本方向，把统筹城乡发展作为根本要求，夯实农业基础，促进农民增收，力争农业增加值增长4%。

积极推进农业结构调整。坚持稳步发展粮食生产，充分发挥粮食主产区的优势，不断提高粮食综合生产能力，力争粮食增产1.5亿斤以上。坚持用工业的理念谋划和发展现代农业，深入实施优质稻产业提升工程、畜牧业振兴工程和水产业壮大工程，加强粮食、畜牧、水产大县（市、区）、大乡（镇）建设，大力发展板块农业、规模农业、特色农业，做大优势产业，提高农产品竞争力和农业综合效益，力争优质稻面积扩大100万亩，生猪出栏增加50万头，水产品产量增加16万吨。坚持扩规模、创品牌，突破性发展农产品加工业，加快形成农产品加工产业集群。积极培育壮大龙头企业，大力发展产业化经营，带动农产品加工、生产基地和农民专业合作组织发展，力争农民专业合作社新增100个。培育壮大农业品牌，推动资源整合和生产标准规范，提高农产品质量，力争新增标志性农产品50个以上。完善“绿色通道”制度，降低农产品运销成本。健全公益性农业技术推广体系，提高农业机械化作业水平，强化重大动物疫病和农作物病虫害防控，增强农业公共服务能力。坚持就地转移、异地输出、自主创业并举，加强农民工就业培训，帮助农民工转移就业和返乡创业，拓宽农民就业渠道和增收空间。

积极推进仙洪新农村试验区建设。抢抓省委、省政府重点建设仙洪新农村试验区的发展机遇，认真编制12个乡镇、336个村的建设发展规划，精心组织实施生产发展、基础设施建设和村庄环境整治等八大工程，努力把仙洪试验区建成深化农村改革的试验区、发展现代农业的示范区、建设社会主义新农村的先行区。强化项目整合和部门联动，继续办好李埠、花基台等新农村示范区和13个省级示范村试点，实施孟溪大垸扶贫开发，支持贫困乡村、革命老区加快发展。加强农村规划管理，大力推进集中居住点建设。实施乡村清洁工程，突出整治村庄环境，切实改善村容村貌。

积极推进农村基础设施建设。加快实施泵站改造、灌区建设等水利工程，鼓励和支持农民广泛开展小型农田水利建设，扎实做好防汛抗灾工作，提高农业防灾减灾能力。统筹建设城乡交通、供电、供水、通信等基础设施，大力推进农村路网、安全饮水、“一建三改”工程建设，推动农村教育、文化、卫生、体育事业发展，努力改善农村生产生活条件。

认真落实强农惠农政策。在稳定农村基本经营制度的同时，积极探索土地承包经营权转包、出租和入股的新路子，引导土地承包经营权向种养大户、农民专业合作社、龙头企业等市场经营主体流转，稳步发展适度规模经营。落实农业补贴和最低收购价政策，落实林业降低育林基金和生产维护费征收标准政策，保护和调动农民生产积极性。严格村级集体资产和财务监管，扶持发展集体经济，探索用改革的办法逐步化解村级债务。严格农民负担预算审批管理，规范 “一事一议”，整治涉农收费行为，防止农民负担反弹。

（四）积极对接鄂西生态文化旅游圈，大力发展现代服务业。坚持市场化、产业化、社会化发展方向，适应扩大内需和消费升级的要求，突出规划引导，不断提高服务业发展水平。

突破性发展文化旅游业。充分发掘楚文化、三国文化、水文化资源优势，加强与鄂西生态文化旅游圈发展规划对接，精心策划、编制和实施文化旅游项目，努力把荆州打造成为鄂西生态文化旅游圈的重要支点和文化中心。完善旅游管理体制和协调机制，加快构建文化旅游开发投融资平台，鼓励国有旅游企业重组发展，大力引进和培育旅游市场主体。狠抓旅游景点景区建设，加快熊家冢古墓保护开发及配套设施、荆州博物馆改扩建和文保中心、游客集散中心、荆州港涉外旅游码头等项目建设，深度开发洪湖岸边是家乡、洈水和天鹅洲等重点景区，推动旅游名镇、名村创建工作，提升旅游产品市场竞争力。积极整合旅游资源，打造特色旅游线路。突出“楚国故都、三国名城”主题，加大宣传营销力度，扩大与张家界、西安等地旅游合作，提升旅游产业整体形象。继续实施荆州古城墙保护维修工程，启动申报世界文化遗产工作。认真做好第三次全国文物普查。鼓励传媒文博、演艺娱乐、创意策划等文化企业加快发展，繁荣文化市场。

做大做强商贸物流业。围绕建设江汉平原商贸物流枢纽城市，认真组织实施城区商业网点规划，优化商业网点布局，调整经营业态，提升服务功能和市场竞争力。规划建设重点物流园区，培育骨干物流企业，推进物流供应链与产业园区对接。落实消费促进政策，提振消费信心，稳定消费预期。完善城市社区便民服务设施，实施“万村千乡市场工程”和“家电下乡工程”，健全农村流通网络，降低消费成本，进一步激活和开拓城乡消费市场。切实加强生产资料、烟草、食品、药品市场监管和价格监督，维护消费者权益，让老百姓放心消费。

（五）深入推进改革开放，增强发展活力。坚持用改革创新的办法破解发展难题，以改革促发展、开放促发展。

进一步深化改革。积极引导国有企业建立规范的现代企业制度，推行国有资本经营预算，实现国有资产保值增值。加快存续企业破产进度，妥善解决资产处置、职工安置等问题，抓紧完成国企改革煞尾任务。认真落实促进非公有制经济发展的相关政策，充分发挥各类商会、协会的桥梁和纽带作用，维护企业合法权益，加快发展个体私营等非公有制经济。继续深化农村综合改革，巩固和完善农村公益性服务“以钱养事”新机制。抓好土地承包经营权流转服务平台建设，有序推进土地流转。稳步开展“小三场”改革，进一步完善国有农场体制改革，基本完成集体林权制度改革任务，积极推进城中村、城郊村、园中村集体资产产权制度创新。支持金融机构拓展支农领域，加快发展政策性农业保险，鼓励发展村镇银行、贷款公司、农村资金互助社等新型农村金融机构，引导更多信贷资金和社会资金投向农村。积极稳妥调整市、区事权关系，强化市级宏观调控职能，完善区级组织经济发展、管理社会事务的能力，逐步形成分工合理、各负其责的管理体制。深化人事制度改革，全面推行事业单位岗位设置管理制度、人员聘用制度。

进一步提高开放水平。强化开放意识，充分挖掘各类优势资源的发展潜力，多领域、全方位引进战略投资，积极拓展利用外资渠道。坚持引资与引智相结合，探索灵活多样的合资合作方式，提高利用外资水平。加快“大通关”建设，确保荆州一类口岸通过升级验收。积极开拓出口市场，努力调整出口产品结构，巩固机电、纺织、化工、轻工等优势产品出口，发展农产品、劳动密集型等优势出口产品，保持出口增长势头。鼓励有条件的企业抓住时机，开展境外经济技术合作。

（六）切实做好财税金融工作，提高促进发展的支撑能力。抢抓国家实施积极财政政策和适度宽松货币政策的机遇，加大增收节支和金融服务力度，努力为经济平稳较快增长提供支持。

加大增收节支力度。大力支持重点企业加快发展，积极促进消费增长，努力挖掘增收潜力，确保财政收入稳定增长。牢固树立过紧日子的思想，优化支出结构，严格控制一般性支出，确保工资发放、机关正常运转、民生改善等支出。强化政府债务管理，积极化解财政风险。加强行政事业单位资产和专项资金监管，完善部门预算、国库集中支付和政府采购等制度，严格执行收支两条线，严肃财经纪律，提高资金使用效益。

提升金融服务水平。积极支持金融机构增加有效信贷投放和资金供应，进一步加强政银企合作，鼓励金融机构简化贷款审批手续，创新服务产品，力争对年销售收入100万元以上有贷款愿望的企业实行信贷全覆盖。广泛开展最佳信用县市区、A级信用企业和信用社区、信用乡镇创建活动，帮助金融机构化解不良资产，努力改善信用环境。扎实抓好新型金融机构建设试点，积极引进股份制银行，扶持小额贷款公司和中小企业担保公司发展。支持市商业银行引进战略投资者和农村信用社达标升级，做大做强地方金融机构。鼓励和指导企业开展上市融资、股权融资、并购融资，拓宽融资渠道。

（七）加强节能减排和生态环境保护，促进发展方式转变。坚持把节能减排和土地集约利用作为转变发展方式的基础工作来抓，推动结构调整升级。

强化节能减排工作。加快重点行业、重点企业技术改造步伐，开发、推广和应用节能减排新技术、新工艺、新产品，优化产业和产品结构。推进纺织印染循环经济工业园和重点企业治污设施建设，强化污染源在线监管，防止发生重大污染事故。加快县市城区、四湖流域重点乡镇污水处理厂建设进度，有效控制环境污染。发挥政府机关示范带动作用，继续开展节能减排全民行动。严格项目环评和节能评估审查，限制新上高能耗、高排放项目。加强节能减排目标评估考核，认真落实问责制和一票否决制。坚持污染治理与生态环境保护并重，切实抓好中心城区水域和洪湖、四湖流域等重点区域的生态修复和建设。

强化土地集约利用。增强节约、集约用地意识，实行最严格的土地管理制度，切实保护好基本农田。积极开展迁村腾地和土地开发整理，挖掘存量和低效土地潜力，保障重大项目用地需求。合理调整用地布局，引导工业企业向开发区（园区）集中，提高单位面积投入强度，有效配置土地资源。严格执行土地出让招拍挂制度，抑制多占、滥占和浪费土地现象。加强土地执法监察，认真清理和处置闲置用地，促进节约集约利用。

四、着力改善民生，切实加强社会建设

坚持把改善民生作为保增长的出发点和落脚点，努力解决人民群众最关心、最直接、最现实的利益问题，让科学发展的成果惠及于民。

切实抓好就业和社会保障工作。以发展促就业、以创业促就业，落实小额贷款、税收优惠、创业指导等政策措施，支持全民创业。积极促进高校毕业生、城镇困难群众、零就业家庭和返乡农民工就业，保持就业稳步增长。进一步做好企业职工、灵活就业人员参加养老保险工作，积极推进被征地农民、农业小三场职工养老保险试点，加快城乡居民医保工作步伐，不断扩大社保覆盖面，确保征收社保资金20.8亿元以上。认真落实企业退休人员基本养老金调标政策，抓紧做好养老保险省级统筹准备，确保社保资金按时发放。进一步完善城乡居民最低生活保障、大病医疗救助和特困户救助制度，切实做好贫困生支助、优抚安置和五保户集中供养等工作。加大廉租房、经济适用房建设投入，逐步解决最低收入家庭住房困难问题。

大力发展各项社会事业。优先发展教育事业，认真落实义务教育学校绩效工资政策，积极化解“普九”债务，巩固“普九”成果；扩大优质高中教育规模，大力发展职业教育，支持长江大学等高校健康发展。高度重视技能型、创新型、管理型人才培养和引进，加大科技推广、成果转化、技术开发公共平台建设力度，推动技术进步和科技创新。深化医药卫生体制改革，完善城乡医疗卫生服务体系，扎实推进血防综合治理工程，加强艾滋病、结核病等重大传染病防治，不断提高公共卫生服务水平。做好人口和计划生育工作，稳定低生育水平。大力弘扬以爱国主义为核心的民族精神和以改革创新为核心的时代精神，积极开展文明城市、文明村镇、文明单位、文明社区创建活动。扎实开展“五五”普法工作，加强未成年人法制教育。推进数字电视整体转换、有线电视村村通和文化惠民工程建设，丰富群众文化生活。加强城乡体育设施建设，精心组织市第四届体育运动会，广泛开展群众性体育活动。完善城乡社区自治和服务功能，加强社团组织登记和管理，发挥工会、共青团、妇联等人民团体在社会服务和管理中的桥梁纽带作用，进一步提高社会管理水平。认真做好拥军优属、拥政爱民工作，积极争创新一轮“双拥”模范城。抓好第六次全国人口普查和国家二类城市语言评估工作。加强外事侨务、台湾事务、民族宗教工作，继续做好气象、地震、移民、档案、史志等工作，重视和支持老龄、残疾人事业发展。

切实维护社会稳定。针对当前社会矛盾风险因素增多的复杂形势，深入推进“平安荆州”建设，健全社会治安防控体系，严厉打击“两抢一盗”等违法犯罪行为，进一步增强群众的安全感。充分发挥行政调解、司法调解和人民调解组织作用，妥善化解人民内部矛盾。继续开展大接访活动，畅通诉求渠道，努力解决群众反映的突出问题。完善应急反应机制，提高预防和处置突发事件的能力。高度重视生产安全和农产品、食品、药品质量安全，落实安全责任，强化隐患整改，严格责任追究，预防重特大安全事故发生。同时，加强国防教育、国防动员、人民防空和预备役、民兵建设等工作。

认真办理十件实事。（1）新增城镇就业人员6.2万人，帮助4200人实现创业并带动1.68万人就业；培训农村劳动力3.5万人，新增转移农村劳动力9.28万人。（2）投资7550万元，新建农村初中学生生活用房10.8万平方米。（3）新建通村公路600公里、农村公路候车亭200个、招呼站300个,完成乡镇渡口达标改造30处。（4）扩大农村低保范围，新增对象2万人，达到16万人；提高农村五保户和农村分散供养孤儿的供养标准。（5）扎实推进血防综合治理，免费救治晚血病人2600人；新建农村户用沼气池3万口，建设乡村沼气服务网点232个和养殖小区联户沼气工程112处；解决农村30万人的饮水安全问题。（6）建设乡镇综合文化站30个，新建或改造农家店400家。（7）为200名贫困白内障患者免费实施复明手术，为100名贫困下肢残疾人免费安装假肢，为1000名贫困精神残疾人免费提供基本控制药物。（8）中心城区筹集廉租住房400套，对居住15平方米以下的低保户和低收入家庭实行租赁补贴；新开工建设经济适用住房5万平方米，启动危旧房改造10万平方米。（9）设立80个社区法律援助接待室，办理法律援助案件900件。（10）改造北京路、红门路等主次干道和管网设施，抓好城市绿化工作，整治城市出入口环境。

五、加强服务型政府建设，不断提高政府工作的能力和水平

扎实开展学习实践科学发展观活动，着力建设“为民、务实、清廉”的服务型政府，努力提高政府执行能力和服务水平。

切实推进依法行政。深入贯彻落实国务院《关于加强市县政府依法行政的决定》，严格按照法定权限和程序履行职责。自觉接受市人大及其常委会的法律监督、工作监督和市政协的民主监督，认真办理人大代表建议和政协委员提案，充分听取各民主党派、工商联和无党派人士的意见，主动接受新闻媒体和社会各界的监督。坚持科学民主决策，健全重大事项集体决策、专家咨询、社会公示与听证制度。抓好电子政务和政府网站建设，完善新闻发布制度，大力推行政务公开，切实保障人民群众的知情权、参与权和监督权。支持审判机关、检察机关依法独立行使审判权、检察权，维护司法公正。支持监察、审计等部门依法履行职责，强化内部监督。改革和完善行政执法体制，探索开展相对集中行政处罚权试点，清理和规范行政处罚自由裁量权，扎实做好行政复议和行政应诉工作，提高依法行政水平。

切实提高政府执行力。深入开展学习实践科学发展观活动，提高领导科学发展、促进社会和谐的能力。加强调查研究，认真分析新情况、新问题，制定新办法、新举措，增强政府工作的预见性、主动性、创造性。牢固树立正确的政绩观，把“创特色、争第一”的要求贯穿到政府工作的各个方面，开拓进取，真抓实干，勇于在困境中找出路、在困难中找办法，把各项工作部署变成实实在在的工作业绩。进一步完善政务督查、绩效考核等抓落实的工作机制，确保政令畅通和各项工作落到实处。

切实优化发展环境。牢固树立亲商、安商、富商意识，把工作重点放在服务项目、服务企业、服务发展上，努力营造让投资者放心、安心、舒心的生产生活环境。按照规矩程序服从于效率的要求，优化管理流程和审批方式，完善首问负责、并联审批、联审联办等制度，增强行政服务中心功能，加强便民服务网络建设，不断提高行政服务质量和水平。严禁以各种名目向企业拉赞助、乱摊派，严禁开展未经批准的检查、评比、培训等活动，严禁向企业指定中介服务机构，从严查处影响投资环境的行为，切实维护投资者和广大人民群众的合法权益。

切实加强廉政建设。积极推进学习型机关建设，提高公务员队伍整体素质。认真落实党风廉政建设责任制，加强对政府工作人员的严格教育、管理和监督。加大源头治腐力度，严格执行工程建设招投标、土地出让招拍挂、政府采购等制度，努力形成靠制度管人制权理事的有效机制。切实加强政风行风建设，坚决纠正有令不行、有禁不止的现象，严肃查处失职、渎职行为。发扬艰苦奋斗、勤俭节约的优良传统，杜绝奢侈浪费，切实降低行政成本。

各位代表，今年的工作任务十分艰巨。让我们紧密团结在以胡锦涛同志为总书记的党中央周围，在省委、省政府和市委的正确领导下，全面贯彻落实科学发展观，振奋精神，锐意进取，开拓创新，扎实工作，努力实现各项目标任务，以优异的成绩迎接建国60周年！
